# Effects of Non-Immersive Virtual Reality Exercise on Self-Reported Pain and Mechanical Hyperalgesia in Older Adults with Knee and Hip Osteoarthritis: A Secondary Analysis of a Randomized Controlled Trial

**DOI:** 10.3390/medicina61071122

**Published:** 2025-06-21

**Authors:** Francisco Guede-Rojas, Cristhian Mendoza, Leonardo Rodríguez-Lagos, Adolfo Soto-Martínez, David Ulloa-Díaz, Carlos Jorquera-Aguilera, Claudio Carvajal-Parodi

**Affiliations:** 1 Exercise and Rehabilitation Sciences Institute, School of Physical Therapy, Faculty of Rehabilitation Sciences, Universidad Andres Bello, Santiago 7591538, Chile; francisco.guede@unab.cl; 2 Escuela de Medicina, Facultad de Medicina, Universidad San Sebastián, Concepción 4030000, Chile; cristhian.mendoza@uss.cl; 3 Cognitive Neuroscience, Pain and Rehabilitation Research Group (NECODOR), Faculty of Health Sciences, Rey Juan Carlos University, 28922 Alcorcón, Spain; rodriguez.lirl@gmail.com; 4 Departamento de Kinesiología, Facultad de Medicina, Universidad de Concepción, Concepción 4030000, Chile; adosoto@udec.cl; 5 Department of Sports Sciences and Physical Conditioning, Universidad Católica de la Santísima Concepción, Concepción 4030000, Chile; dulloa@ucsc.cl; 6 Escuela de Nutrición y Dietética, Facultad de Ciencias, Universidad Mayor, Santiago 8580745, Chile; carlos.jorquera@mayor.cl; 7 Escuela de Kinesiología, Facultad de Ciencias de la Rehabilitación y Calidad de Vida, Universidad San Sebastián, Concepción 4030000, Chile

**Keywords:** virtual reality, osteoarthritis, older adults, pain, Visual Analog Scale, pressure pain threshold, MCID

## Abstract

*Background and Objectives*: Osteoarthritis (OA) of the knee and hip is a major cause of pain and functional impairment. This study evaluated the effects of non-immersive virtual reality (NIVR) combined with conventional physical therapy (CPT) on pain intensity, mechanical hyperalgesia, and perceived recovery in older adults with OA. *Materials and Methods*: Sixty older adults with mild-to-moderate knee or hip OA were randomly assigned to a NIVR group (NIVR-G; *n* = 30) or a CPT group (CPT-G; *n* = 30). Both groups completed 30 sessions over 10 weeks (3 sessions/week). The NIVR-G performed 20 minutes of exergames integrated into CPT. Pain intensity was assessed using the Visual Analog Scale (VAS), and mechanical hyperalgesia was evaluated through pressure pain thresholds (PPTs). Secondary outcomes included the Global Rating of Change (GRoC) and the minimal clinically important difference (MCID) for the VAS. This study is a secondary analysis of a randomized controlled trial registered at ClinicalTrials.gov (ID: NCT05839262). *Results*: The NIVR-G demonstrated significant reductions in pain intensity after 30 sessions (*p* < 0.05, *d* = 1.50), with greater improvements compared to the CPT-G (*p* < 0.05, *d* = 1.17). The MCID for the VAS was established at 9.2 mm, with a higher proportion of responders in the NIVR-G (*p* < 0.05). The NIVR-G also reported superior recovery perception on the GRoC scale (*p* < 0.05). No significant changes in PPTs were observed in either group. However, the improvements in the NIVR-G diminished four weeks post-intervention. *Conclusions*: NIVR exergames combined with CPT significantly reduced pain intensity, improved perceived recovery, and resulted in a higher proportion of responders compared to CPT alone. These findings support the use of NIVR as an effective adjunct to CPT in older adults with OA; however, further research is needed to optimize its long-term benefits.

## 1. Introduction

Knee and hip osteoarthritis (OA) are prevalent degenerative joint diseases that significantly impact global health, affecting over 300 million people and ranking as a leading cause of disability worldwide [1,2]. Knee OA has a global prevalence of approximately 3.8%, while hip OA affects around 0.85% of the population [3]. The disease burden increases with age, affecting up to 13% of women and 10% of men over 60 [4]. Key risk factors for OA include age, female sex, obesity, joint injuries, and genetic predisposition [1,2]. Rising life expectancy and obesity rates are projected to further increase OA prevalence, emphasizing the need for public health interventions and healthcare preparedness [2,5].

Pain is the most common symptom in knee and hip OA, involving a range of mechanisms and interactions, and is associated with deterioration in physical function, future disability, and a significant impact on patients’ quality of life [1,6]. OA pain typically presents as a dull, aching discomfort that becomes more persistent over time, with intermittent episodes of intense and unpredictable pain, disrupting social and recreational activities [7]. Pain perception is influenced by both age and sex, with younger patients reporting higher pain intensity and using more affective descriptors despite having less severe disease, while women tend to report more pain and use more analgesics compared to men [8]. Radiographic severity does not always correlate with pain intensity, particularly in hip OA, where pain can be more severe despite less advanced radiographic changes [9,10]. Therefore, comprehensive pain management strategies should address the multifaceted nature of OA pain to improve patient outcomes.

In OA, pain is driven not only by joint pathology but also by altered nociceptive processing, such as mechanical hyperalgesia [11]. Patients with knee OA, for example, exhibit lower pressure pain thresholds (PPTs) not only at the knee but also at remote sites, suggesting widespread hyperalgesia [12]. This phenomenon has been described as pain sensitization and could imply the presence of central sensitization (CS), where the central nervous system amplifies pain signals, lowering pain thresholds throughout the body [13,14,15]. This is consistent with findings from other chronic musculoskeletal pain conditions, supporting the idea that OA pain extends beyond the affected joint [16]. CS has been proposed as one of the mechanisms underlying the lack of correlation with structural damage observed in imaging studies [17]. Furthermore, neuropathic pain may be present in OA patients, being more common in knee OA than in hip OA, with up to 40% of knee OA patients affected [13]. As such, pain management for OA must address both local and central mechanisms to improve pain control and enhance overall patient outcomes [12].

The management of knee and hip OA involves a combination of non-pharmacologic, pharmacologic, and surgical treatments tailored to the severity of the disease and individual patient needs, with non-pharmacologic treatments being foundational, and exercise and weight loss as key components [18]. Exercise, including both aerobic and resistance training, is highly recommended, as it improves pain and function without significant adverse effects [19]. In addition to exercise, common self-management strategies such as the use of knee braces or supports have shown efficacy in symptom relief and functional improvement [20]. Other non-pharmacologic therapies, including physical modalities, life-style modification, and patient education, also contribute to comprehensive OA management [21]. Pharmacologic treatments, such as nonsteroidal anti-inflammatory drugs, serve as first-line options for pain relief, intra-articular corticosteroid injections offer short-term pain management for both knee and hip OA, and for advanced OA that does not respond to conservative treatments, total joint arthroplasty is the most effective surgical intervention [19,22]. However, many patients continue to experience persistent pain and functional limitations after conventional treatments, which has led to the exploration of new therapeutic approaches.

Virtual reality (VR), defined as the creation of digital environments that allow interaction, can be classified based on its level of immersion into fully immersive, semi-immersive, and non-immersive [23]. Physical exercise performed through non-immersive virtual reality (NIVR) systems (also known as exergames) and delivered either as a standalone intervention or combined with conventional physical therapy (CPT) has emerged as a promising tool for managing pain and improving various outcomes in individuals with OA, including postural balance, proprioception, gait, range of motion, fall risk, depression, and quality of life [24,25,26,27]. Several studies have specifically examined the effects of NIVR interventions on pain reduction, yielding encouraging results as measured by instruments such as the Western Ontario and McMaster Universities Osteoarthritis Index (WOMAC) and the Visual Analog Scale (VAS) in patients with knee OA [24,25,26,28]. Additionally, VR rehabilitation has demonstrated potential in reducing kinesiophobia and pain catastrophizing in patients undergoing total knee arthroplasty [29]. Despite these positive findings, the effectiveness of exergames relative to conventional rehabilitation remains unclear, and more high-quality studies are needed to establish definitive clinical guidelines [25,30]. In summary, NIVR shows promise as a complementary approach to OA pain rehabilitation, though further research is required to refine and standardize its clinical application.

VR systems seem to reduce pain through a combination of psychological and physiological mechanisms. One key mechanism is distraction and immersion, where the multisensory nature of VR diverts attention from pain, lowering its intensity and unpleasantness [31]. Research shows that immersive VR environments, especially those involving interactive elements like avatar embodiment, effectively reduce pain perception [32,33]. Additionally, physical activity during exergaming induces hypoalgesic effects [34]. Active VR games that require movement have been shown to increase pain thresholds and reduce sensitivity, particularly when they elicit cardiovascular responses [35,36]. Neural mechanisms also play a role, with VR altering neural oscillations, such as gamma and alpha waves, which are linked to reduced pain intensity [33]. Furthermore, tactile feedback enhances the sense of avatar ownership, which increases VR’s effectiveness in alleviating pain [37]. These mechanisms together make NIVR a promising non-pharmacological approach to pain management [25].

Despite promising findings, a comprehensive understanding of the mechanisms through which NIVR modulates pain and influences pain perception is still lacking, requiring further investigation [31]. Moreover, additional controlled trials comparing NIVR with conventional interventions are needed to definitively establish its efficacy in pain management [25,38], which could contribute to developing more personalized and effective treatment strategies in older adults with knee and hip OA [24,25,28,31]. Furthermore, to the authors’ knowledge, no studies have yet examined the effect of NIVR on clinically meaningful measures, such as the minimum clinically important difference (MCID) and the Global Rating of Change (GRoC) scale for pain in this population. Therefore, this study aimed to evaluate the effect of an NIVR intervention on self-reported pain intensity and the response of local and remote mechanical hyperalgesia in patients with knee and/or hip OA. We hypothesized that NIVR exercise combined with CPT would reduce self-reported pain intensity and mechanical hyperalgesia in patients with knee and/or hip OA. Secondarily, an aim was to establish the MCID threshold for the VAS to identify responders and non-responders to the NIVR treatment in terms of pain intensity.

## 2. Materials and Methods

### 2.1. Study Overview

This study was a secondary analysis of a parallel, two-arm randomized controlled trial with a 1:1 allocation [39]. The experimental group (NIVR-G) engaged in NIVR complementarily to CPT, while the control group (CPT-G) followed the CPT protocol exclusively. The protocol received ethical approval from the Scientific Ethics Committee of the Concepción Health Service (Approval No. 22-12-59) and was registered at clinicaltrials.gov (NCT05839262) on 15 March 2023.

### 2.2. Participant Recruitment

The participants were selected from Lorenzo Arenas Primary Health Care Center, Bíobío, Chile. A recruitment process was conducted by a professional who contacted eligible patients referred for physical therapy, either in person or by phone. Those expressing interest attended a briefing session where the study’s details were explained, and written informed consent was obtained in accordance with the Declaration of Helsinki.

**Figure 1:**
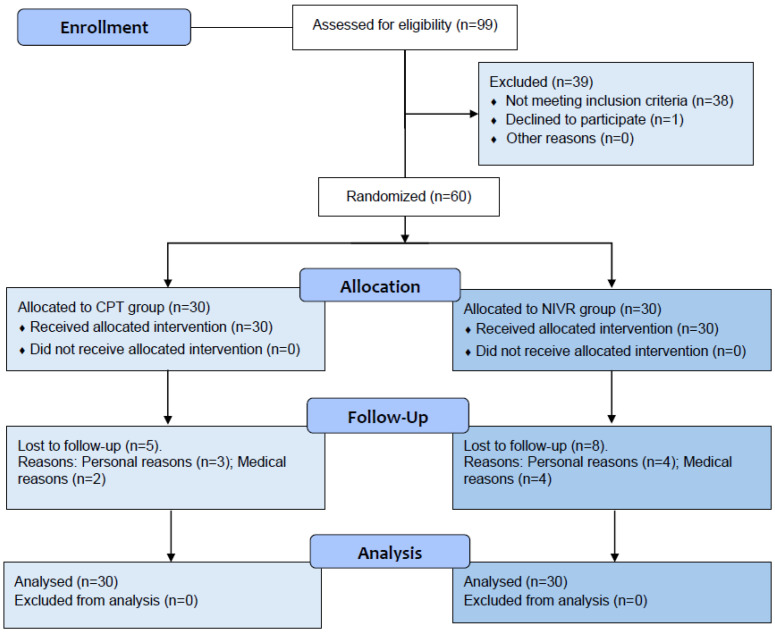
Study flow chart.

Demographic and clinical data were collected during the initial session (Table 1), and the participants were introduced to the study procedures to ensure familiarity. The inclusion criteria encompassed adults aged 60–84 years with mild-to-moderate knee or hip OA diagnosed using the American College of Rheumatology (ACR) criteria and graded 2–3 on the Kellgren–Lawrence scale [40,41]. The exclusion criteria included uncontrolled physical or cognitive conditions, opioid use, scores below 13 on the abbreviated Mini-Mental State Examination (MMSE-EFAM) [42], OA from infectious or autoimmune causes, recent surgeries or fractures, and participation in other rehabilitation programs within the past three months. The participant flow throughout the study phases is shown in Figure 1.

Sample size calculations were conducted using G*Power (v3.1.9.7), targeting a moderate effect size (α = 0.05, power = 0.8). To account for potential attrition, 60 participants were recruited, exceeding the estimated 50 required.

### 2.3. Group Allocation

The participants were randomized by an independent researcher external to the research group, using a stratified randomization strategy in R software (v4.1.2), ensuring balance by age and sex. Group assignments were concealed in sequentially numbered, opaque envelopes, which were opened by a healthcare professional at the time of allocation.

### 2.4. Intervention Protocol

The 10-week intervention consisted of three supervised sessions per week for both groups, totaling 30 sessions. Each session included 50 min of effective exercise time. Adherence was defined as attendance at a minimum of 20 sessions. Exercise intensity was monitored using a 0–10 perceived exertion scale, with the participants instructed to maintain a light (ratings of 3–4) to moderate (ratings of 5–6) effort. The intervention was designed to meet a target weekly exercise volume of 150 min, in alignment with the recommendations of the American College of Sports Medicine (ACSM) [43].

**Figure 2:**
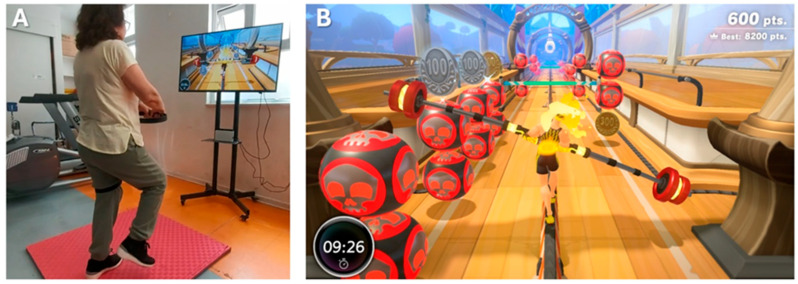
Experimental group subject performing NIVR intervention (**A**) and their avatar for feedback (**B**).

- Control group: CPT sessions included physical agents (e.g., TENS, hot packs), warm-up, structured exercises (aerobics, strength, balance, and flexibility), and a cool-down. Individualized adaptations ensured the exercises matched participants’ capacities.
- Experimental group: CPT was complemented with 20 min of NIVR, replacing part of the CPT exercise block to maintain an equal session duration of 50 min. The NIVR included activities targeting strength, balance, flexibility, and aerobic endurance, delivered through Ring Fit Adventure (Nintendo Switch_®_, Nintendo Co., Ltd., Kyoto, Japan) on a 43-inch TV with real-time feedback (Figure 2). Specifically, the exercises performed using the NIVR device were as follows: Dorsal rotation, Rotation with inclination, Knee raises, Squats, Lunge with rotation, Lateral inclination, Squats with extension, The warrior, The chair, Crescent moon, Equilibrium, Moto adductors, Trunk swinging, Running path, Monster’s lair, and Jogging bridge.

### 2.5. Outcome Assessments

A trained health professional, blinded to the group assignment, conducted assessments at five time points: baseline (one week prior to the start of the intervention), after 10 sessions, after 20 sessions, after 30 sessions, and at follow-up four weeks after the intervention ended.

Pain intensity was measured using the VAS, a widely recognized and reliable tool. The participants were asked to rate their actual pain intensity on a scale from 0 (no pain) to 10 (worst possible pain) [44].

The PPT, a reliable and reproducible measurement tool [45,46], was used to assess the activation of endogenous analgesic mechanisms and mechanical hyperalgesia, a marker of generalized mechanical pain sensitization. These measurements were taken at three anatomical sites: the upper trapezius muscle (PPT-T, representing remote or widespread hyperalgesia), the knee (PPT-K, midway between the medial epicondyle and the patellar tendon at the joint line), and the greater trochanter of the hip (PPT-H). The assessments followed validated protocols [47,48] and used a Wagner FPX 25 digital algometer (Wagner Instruments, Greenwich, CT, USA). Measurements were performed on the affected side or the dominant side in cases of bilateral involvement. The evaluator, consistent throughout the study, applied the algometer perpendicularly to the skin at a standardized pressure increase rate (0.5 kg/cm^2^ per second) until the participant reported pain onset. Each site was measured three times, with a three-minute rest between repetitions. The mean value from the three measurements at each site was used for the analysis.

Changes in perceived pain were assessed solely at the final evaluation (after 30 sessions) using the GRoC scale. This 15-point scale, commonly used as both an outcome measure and an external anchor, ranges from +7 (“a very great deal better”) to −7 (“a very great deal worse”), with 0 indicating no change. The change was categorized as small (1–3 points), moderate (4–5 points), or large (6–7 points) [49].

### 2.6. Statistical Anlysis

The data were analyzed using an intention-to-treat approach. This analysis was conducted by an investigator who was blinded to the group allocation. Missing values were managed with predictive mean matching multiple imputation [50]. Normality was assessed using the Shapiro–Wilk test, and homoscedasticity was evaluated with Levene’s test. A significance level of *p* < 0.05 was applied to all analyses. A two-way repeated measures analysis of variance (ANOVA), followed by Bonferroni post-hoc tests, was performed for the VAS and PPT scores. To address unequal variances between groups (as assessed by the Brown–Forsythe test, *p* < 0.05), the Welch test was applied to evaluate differences in the GRoC of pain. Effect sizes were calculated using Cohen’s *d* and categorized as negligible (<0.2), small (0.2–0.49), moderate (0.5–0.79), or large (≥0.8) [51].

For the calculation of the MCID, the anchor-based method using the GRoC was employed. The patients were categorized according to their score on this scale as follows: (i) No change (GRoC = −1, 0, and 1), (ii) Minimal change (GRoC = −2, −3, 2, and 3), (iii) Moderate change (GRoC = −4, −5, 4, and 5), (iv) Large change (GRoC = −6, −7, 6, and 7). Subsequently, the absolute mean changes in the VAS scores were calculated based on the change perceived by the patient using the GRoC scale. To identify responders and non-responders, the MCID threshold for the VAS was calculated using a standardized effect size multiplied by the standard deviation [52]. This method accounts for inter-individual variability and allows for the identification of responders whose improvements exceed the MCID, reflecting clinically meaningful benefits. The participants were classified based on the change (Δ) in scores (post-test 3 vs. pre-test) into two groups: responders (Rs) and non-responders (NRs) [53]. The classification criteria were defined as follows: Rs if Δ score ≥ MCID, and NRs if Δ score < MCID.

## 3. Results

At the beginning of the study, the groups exhibited comparable sociodemographic characteristics (Table 1) and baseline levels of the study outcomes. Of the 99 individuals initially screened, 60 participants were enrolled, with 13 dropouts for reasons unrelated to the study protocol (Figure 1). Recruitment and follow-up occurred between April 2023 and March 2024. No adverse events, such as falls, fainting, nausea, or severe pain, were reported. Adherence rates were 73.3% in the CPT-G and 76.7% in the NIVR-G.

**Figure 3:**
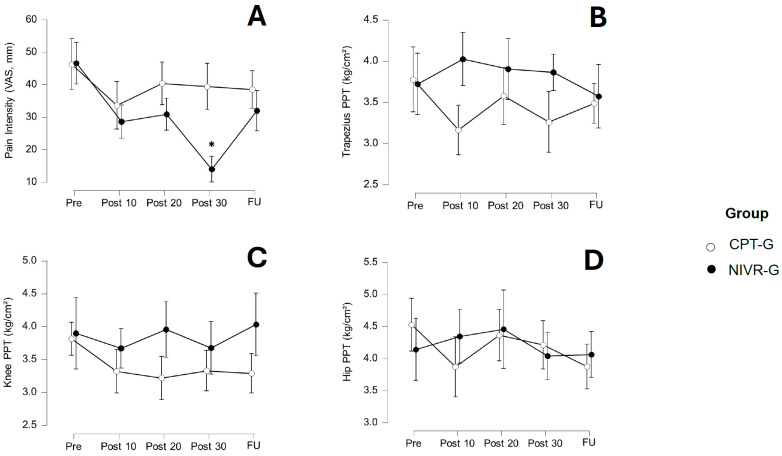
Changes in pain intensity (**A**) Visual Analog Scale [VAS], trapezius pressure pain threshold [PPT] (**B**), knee PPT (**C**), and hip PPT (**D**) after 10, 20, and 30 sessions, as well as at follow-up (FU), in the conventional physical therapy group (CPT-G) and the non-immersive virtual reality group (NIVR-G). The means and 95% confidence intervals are shown for each group at each time point. An asterisk (*) indicates a statistically significant difference between groups, in favor of the NIVR-G.

The VAS, PPT-T, PPT-K, and PPT-H values are presented in Table 2. For VAS pain intensity (Figure 3A), the within-subjects ANOVA revealed significant changes over time (*p* < 0.001) and a significant interaction between time and group (*p* < 0.001). The between-group comparisons indicated a significant difference between the NIVR-G and CPT-G (*p* = 0.029). The post-hoc intragroup analysis showed no significant differences in pain intensity over time within the CPT-G. However, the NIVR-G demonstrated significant differences after 10 (*p* < 0.05; mean difference [MD] = 18.00; 95% CI [3.89–32.11]) and 30 sessions (*p* < 0.05; MD = 32.63; 95% CI [17.04–48.23]), with large effect sizes (*d* = 0.825, and 1.496, respectively). The post-hoc comparisons between the groups showed that only after 30 sessions did the NIVR-G demonstrate a significant reduction in pain intensity compared to the CPT-G (*p* < 0.001; MD = 25.43; 95% CI [12.83–38.03]), with a large effect size (*d* = 1.166).

The within-subjects ANOVA for the PPT-T revealed no significant main effect of time (*p* = 0.540), though the interaction between time and group was significant (*p* = 0.039). However, the post-hoc Bonferroni’s tests did not show significant differences between the comparisons. The between-subjects analysis also did not find a significant group difference (*p* = 0.156). Figure 3B illustrates the changes in these variables after both interventions.

For the PPT-K (Figure 3C), the within-subjects ANOVA indicated no significant main effect of time (*p* = 0.267), and the interaction between time and group was not significant (*p* = 0.311). The between-subjects analysis revealed no significant group difference for the PPT-K (*p* = 0.069), suggesting no overall differences between the groups in PPT-K scores.

For the PPT-H, no significant main effect of time was observed (*p* = 0.223). Similarly, the interaction between time and group was non-significant (*p* = 0.296), and the between-subjects analysis found no significant group differences (*p* = 0.901). These results suggest that the intervention had no significant impact on the PPT-H, either across time or between the groups (Figure 3D).

**Figure 4:**
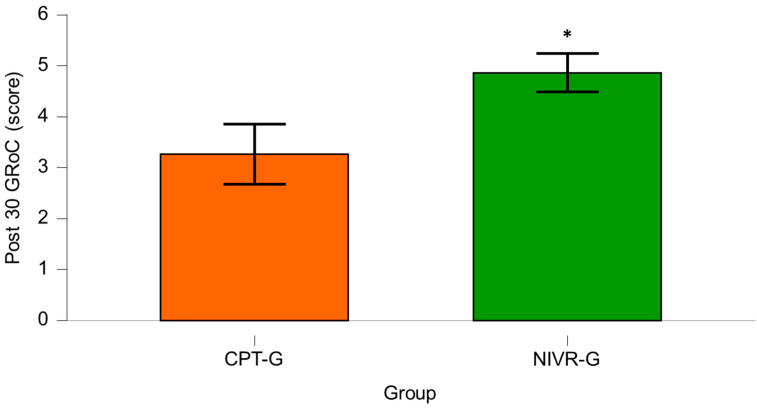
Comparison of Global Rating of Change for Pain (GRoC) after 30 sessions in the conventional physical therapy group (CPT-G) and the non-immersive virtual reality group (NIVR-G). An asterisk (*) indicates a statistically significant difference in favor of the NIVR-G.

Finally, after 30 sessions, the GRoC scores for the CPT-G showed a mean of 3.27 ± 1.57, while the NIVR-G exhibited a mean score of 4.87 ± 1.01. A statistically significant difference was found between the groups (*p* < 0.001; MD = 1.60; 95% CI [0.91–2.29]), with a large effect size (Cohen’s *d*= −1.210), indicating a meaningful difference in favor of NIVR-G (Figure 4).

The Supplementary Material 1 (Table S1) presents the absolute mean changes (95% CI) in the VAS scores based on the patient’s perceived change. The anchor-based estimation of the MCID for VAS was 9.2 mm.

The Supplementary Material 2 (Figure S1) presents the ±MCID thresholds for the VAS scores, along with bars representing each participant’s Δ scores for their classification as Rs and NRs. In the NIVR-G, the most frequent category was Rs, whereas in the CPT-G, both categories were equivalent. Compared to the CPT-G, the NIVR-G had higher frequencies of Rs (76.67%), with a statistically significant difference in the VAS (chi-square test, *p* < 0.05; z-test, *p* < 0.05). For the NRs category, the NVIR-G had lower frequencies, with a statistically significant difference in the VAS (chi-square test, *p* < 0.05; z-test, *p* < 0.05).

## 4. Discussion

The results suggest that adding NIVR enhances the effect of CPT on pain intensity in older adults with hip or knee OA after 30 sessions. Furthermore, this aligned with the patients’ perceptions of improvement in their pain condition following the intervention. However, this improvement was not maintained at the 4-week follow-up. Additionally, the results show that local and remote PPTs did not significantly change after 10, 20, or 30 sessions, nor at the 4-week post-intervention follow-up. Therefore, CPT, whether complemented with NIVR or not, did not significantly alter local or remote mechanical hyperalgesia in the sample studied. Finally, the MCID for VAS after 30 sessions was estimated at 9.2 mm for this population.

### 4.1. Self-Reported Pain Intensity

The findings of this study regarding pain intensity reduction are aligned with previous research on the effects of rehabilitation using various VR modalities. These include studies on knee and hip OA, as well as other chronic painful conditions [24,54,55]. Additionally, the lack of long-term results observed in the present study is consistent with previous reports by Mallari et al. [56]. A recent systematic review showed that both NIVR and immersive VR training are effective for alleviating chronic musculoskeletal pain [57]. Specifically for knee OA, two recent reviews concluded that VR-based exercise therapy is effective in improving pain [25,28]. Wei et al. [28] analyzed nine studies, five of which used NIVR for 4 to 12 weeks, with a frequency of 2 or 3 times per week. Subgroup analysis based on the VR immersion level showed that immersive VR was more effective at relieving pain compared to NIVR, which could be attributed to a lower level of sensory substitution in NIVR, leading to a reduced capacity to attenuate pain perception. On the other hand, in their review on the effects of exergames in patients with OA, Guede-Rojas et al. [25] found that in three out of four studies reviewed, NIVR yielded better results than the control group in reducing pain intensity in knee OA, which is consistent with our findings after 30 sessions. Finally, although VR interventions have the potential to reduce attention to pain and improve pain perception by stimulating visual, auditory, sensorimotor, cognitive, and affective networks [31,58], further clinical studies are required to elucidate the mechanisms underlying our findings, specifically, the mechanisms involved in pain intensity reduction in individuals with OA after completing a NIVR protocol and the reasons why these improvements do not persist over time.

Regarding treatment duration, the systematic review and meta-analysis by Wei et al. concluded that treatments lasting less than 6 weeks are more effective than those lasting 6 weeks or longer [28]. Furthermore, the best results are observed with treatment frequencies of ≤3 times per week and session durations of 20 min each [28]. Despite these reported values, our study demonstrated significant results with a large effect size when complementing CPT with NIVR applied at a frequency of 3 times per week over a 10-week period, with a session duration of 20 min, immediately following the completion of the treatment. These differences could be attributed to the characteristics of the devices used, the type of exercise performed, the protocol parameters, or the level of immersion in the interventions.

Besides its therapeutic utility on its own, adding NIVR to CPT appears to provide greater short-term benefits for pain intensity compared to CPT alone in patients with hip or knee OA in the short term. The combination of locally applied interventions (e.g., exercises) and centrally-targeted interventions (e.g., NIVR) may produce synergistic effects, resulting in better outcomes [28,31,38,58]. Several studies have reported that adding a new therapeutic tool to CPT could improve the outcomes achieved. For instance, a recent review found low-to-moderate-certainty evidence supporting manual therapy as an adjunct to exercise therapy for pain in patients with knee or hip OA in the short term [59]. However, the effectiveness of other tools, such as bracing [60], orthotics [61] and dry needling [62], as part of a multidisciplinary approach, remains questionable. The results of this clinical trial support the idea of complementing CPT with tools like NIVR. However, further studies are needed to better characterize the interventions and patients who would benefit from this therapeutic synergy, especially regarding long-term outcomes.

The severity of reported pain intensity has been associated with the degree of pain sensitization, and this association is independent of the radiologically-reported severity of OA [14]. This gives significant clinical relevance to our NIVR results, particularly considering that acute OA flare-ups can reduce adherence to active exercise treatment [63,64]. In our study, the additional improvement in pain intensity offered by NIVR occurred after approximately 30 sessions, suggesting that NIVR would not be the first choice for the rapid management of acute OA flare-ups. This does not mean that NIVR cannot help improve patient adherence to treatment, as it may be the most motivating way for some individuals to engage in exercise [65]. OA is a heterogeneous disease, so subgroups of patients may respond differently to various treatment modalities. In this regard, clinicians must consider the patient’s perspective when setting therapeutic goals [66], as these may differ from the goals of a study group (e.g., the mean change in a variable). This supports the use of clinical measurements in such studies, and in this sense, the differences observed between the groups for the post-treatment GRoC variable—positive in favor of the NIVR-G—suggest recommending its use both in clinical practice and in outcome assessments in future studies.

Most studies on OA have primarily focused on short-term outcomes, leaving the long-term effects of VR-based therapies insufficiently explored [28]. In our study, although the combination of NIVR with CPT produced significant reductions in pain intensity after 30 sessions—with large between-group effect sizes—these benefits were not maintained at the 4-week follow-up. Similar patterns have been observed in previous research on exercise programs based on other digital technologies (such as mobile applications or telerehabilitation), where clinical improvements tend to diminish over the long-term following treatment cessation [67]. This suggests that ongoing engagement or booster sessions may be necessary to sustain therapeutic gains. Future studies should investigate the feasibility and efficacy of reinforcement strategies or home-based NIVR programs to improve the long-term management of knee OA.

### 4.2. Mechanical Hyperalgesia

Patients with different types of OA may exhibit pain sensitization, characterized—among other variables—by a decrease in baseline PPTs, both locally (peripheral sensitization) and remotely (central sensitization) [68]. Specifically, in patients with knee and hip OA, a robust body of literature has shown the presence of both local and remote mechanical hyperalgesia when compared to healthy controls [11,12,14,69,70,71,72], which is related to functionality levels [73]. Increased mechanical pain sensitization is a pre-morbid risk factor for worsening knee and hip OA symptoms [74,75,76], and a predictor of poor treatment response [11,76]. Interestingly, several studies have reported that alterations in PPTs can normalize once the nociceptive source is surgically treated [77,78,79], while Hattori et al. reported that therapeutic exercise may reduce pain intensity in people with knee or hip OA, depending on baseline symptom severity and mechanical hyperalgesia [11]. Fingleton et al. observed that PPTs’ responses to exercise in people with OA are related to conditioned pain modulation (CPM) test behavior, suggesting that exercise and CPM share equivalent mechanisms [80]. Specifically, they found that individuals with normal CPM showed improvements in their PPTs, whereas those with impaired CPM exhibited decreased PPTs. In contrast, our study found no significant changes in local and remote PPTs values following the intervention.

The baseline PPTs obtained in our sample were similar to those reported in previous studies for individuals with knee or hip OA; moreover, they were more than 20% lower than those measured in healthy individuals [73,81,82]. This clinically significant difference indicates the presence of both local and remote mechanical hyperalgesia [14,83,84]. Remote mechanical hyperalgesia has been described as an indicator of generalized pain sensitization [12] and alterations in nociceptive processing, which are associated with poor responses to standard treatment in OA patients [85]. This highlights the therapeutic potential of the descending modulatory system to influence pain thresholds. This system works by releasing neurotransmitters from the brain that can either inhibit or enhance nociceptive transmission in the spinal cord [86]. Thus, emotional states and sensory experiences can influence pain perception through limbic and thalamic regions of the brain.

Based on the reasoning outlined above, it is important to highlight that this study observed a paradoxical finding: adding NIVR to the standard treatment did not modify local or remote mechanical hyperalgesia, but it did significantly reduce the pain intensity perceived after 30 sessions of intervention. The cognitive-emotional stimuli associated with VR exposure, such as distraction, affective response, ritualization, positive experience, learning through exposure, and anxiety control, may have acted as the main modulators of the analgesic response perceived by the patients at the end of the intervention [25,31]. NIVR has been shown to induce improvements in various positive emotions, such as happiness, self-esteem, self-worth, self-efficacy, vitality, motivation, and relaxation [87], which could have facilitated the attenuation of the self-reported pain perception through mechanisms such as distraction and activation of motivational systems that counteract the perception of pain [88]. Additionally, dysfunction in exercise-induced analgesic mechanisms has been reported in individuals with chronic pain and pain sensitization [11,89,90], which, according to Previtali et al., may be present in up to one-fifth of patients with knee OA [71]. As mentioned, this dysfunction is reflected in increased pain intensity and duration, influenced by factors such as emotions, stress, and cognitive processes. However, although the presence of sensitization has been identified in individuals with knee or hip OA, the reasons why some patients experience pain only associated with activity, while others suffer from persistent pain, remain unknown.

It is tempting to speculate that new VR modalities may more effectively activate descending inhibitory pathways through distraction with cognitively demanding tasks. However, our study did not show a sustained improvement in pain intensity when assessed 4 weeks post-intervention. Future studies should examine whether VR can achieve this effect and what conditions are required to facilitate it. Additionally, to increase clinical relevance and measurement accuracy, it is suggested that future research include CPM and temporal summation. These are dynamic tests used to investigate central pain processing by assessing an individual’s response to experimental pain stimuli [91]. These quantitative sensory tests, along with PPTs, are the most commonly used measures to assess pain sensitization and may contribute to identifying individuals at risk of chronic pain, guiding a more personalized and effective treatment for OA [92]. Finally, although NIVR showed benefits in pain intensity perception, the protocol may not have been sufficient or optimal to induce significant changes in PPTs, considering that the appropriate parameters for prescribing exercise to improve pain sensitization in chronic pain populations, including OA, are still unknown [93,94].

### 4.3. Minimal Clinically Important Difference

Research on the MCID for pain intensity in OA has provided valuable insights into interpreting treatment outcomes. For knee and hip OA, a reduction of 1 point or 15% on the numerical rating scale is considered clinically important. Additionally, changes of 19.9 mm for knee OA and 15.3 mm for hip OA on the VAS are also recognized as clinically meaningful improvements [95,96]. In our study, we estimated the MCID for pain intensity (VAS) at 9.2 mm. This value serves as an important benchmark for assessing clinically relevant pain reductions in OA patients undergoing NIVR in conjunction with CPT. The discrepancy between our findings and previous research may be attributed to differences in sample characteristics, including factors such as baseline symptom severity, age, sex distribution, and the specific treatment applied. These factors can significantly influence the achievement of MCID, with greater improvements typically observed in younger patients, females, and those with more severe baseline symptoms [97].

### 4.4. Strengths and Limitations

This study has several limitations that should be considered when interpreting the results. First, the follow-up period post-treatment was limited to one month, which prevents an assessment of the long-term effects of NIVR on pain intensity and mechanical hyperalgesia. Additionally, the measurement of PPTs can be challenging in certain patients due to the difficulty in accurately identifying the transition point between pressure and perceived pain [98]. Another notable aspect is that the sample, although calculated using G*Power (α = 0.05, power = 0.8), was relatively small and included individuals with both unilateral and bilateral hip and knee OA, as well as exclusively mild-to-moderate disease severity. These clinical characteristics may have influenced individual responses to the intervention and limit the generalizability of the findings to patients with more advanced OA or more uniform clinical profiles. Moreover, the predominance of female participants and minor imbalances in baseline characteristics—such as educational level and OA location—could also have affected pain perception or treatment response. Therefore, the findings should be interpreted with caution and may not be generalizable to the broader OA population. Finally, although both groups received equivalent total exercise time and matched intensity levels, the substitution of 20 min of CPT with NIVR may limit the interpretation of the additive effects of VR. These limitations highlight the need for future studies with larger sample sizes, extended follow-up periods, and multi-arm designs that allow for isolating the specific contribution of NIVR in addition to full-dose conventional physiotherapy.

The strengths of this study include its randomized controlled design, recognized as the gold standard in clinical research, which enhances internal validity and minimizes the risk of bias. Additionally, strategies such as stratified randomization and concealed allocation were implemented, ensuring balance between the groups and reducing the possibility of bias in participant assignment. The pragmatic application of the protocol in a real-world clinical setting strengthens the external validity of the findings, facilitating their extrapolation to similar clinical contexts. Moreover, established, validated, and reliable measurement tools, such as the VAS and PPTs, were used. The inclusion of clinically significant outcome measures, such as the MCID and GRoC, emphasizes the patient-centered approach and reinforces the applicability of the findings. On the other hand, the comparison between groups was appropriate due to the equivalent weekly volume of physical exercise, which allowed for the evaluation of the effects of the interventions without introducing confounding by differences in total physical activity load. Finally, the study considered an intention-to-treat analysis, ensuring that the results reflect the effect of the intervention in the originally assigned population, thereby reducing the influence of potential biases due to data loss during the intervention.

## 5. Conclusions

The application of 30 sessions of NIVR combined with CPT in older adults with knee and hip OA resulted in significant short-term improvements in pain intensity and global perceived recovery (GRoC). However, no changes were observed in local or remote PPTs. Additionally, pain intensity improvements were not sustained, as they reverted one month after the intervention. These findings suggest that while NIVR can enhance the effects of CPT on subjective pain and recovery during the intervention, its impact may be transient. Moreover, the lack of effect on mechanical hyperalgesia highlights the need for further research to develop strategies that promote long-term pain relief and address the underlying mechanisms of pain sensitization in this population. Future studies should also explore the key characteristics of intervention protocols that drive these analgesic responses.

## Figures and Tables

**Table 1:** Sociodemographic characterization of the sample.

	CPT-G (*n* = 30)	NIVR-G (*n* = 30)	*p*-Value
Age (years), M ± SD	69.00 ± 5.56	68.73 ± 5.48	0.852
BMI (kg/m^2^), M ± SD	30.17 ± 4.35	29.83 ± 4.44	0.761
Number of drugs, M ± SD	2.53 ± 1.50	2.37 ± 1.56	0.675
Number of comorbidities, M ± SD	1.23 ± 0.86	1.27 ± 0.98	0.889
Sex (female/male), *n*	25/5	25/5	1.000
Educational level			
Primary, *n* (%)	6 (20.00%)	6 (20.00%)	1.000
Secondary, *n* (%)	19 (63.33%)	12 (40.00%)	0.071
Incomplete higher education, *n* (%)	2 (6.67%)	3 (10.00%)	0.638
Professionals, *n* (%)	3 (10.00%)	9 (30.00%)	0.052
Osteoarthritis diagnosis, *n* (%)			
Knee	15 (50.00%)	19 (63.33%)	0.298
Hip	6 (20.00%)	7 (23.33%)	0.756
Knee + Hip	9 (30.00%)	4 (13.33%)	0.116

CPT-G control group; NIVR-G experimental group; M mean; SD standard deviation; *n* number; BMI body mass index.

**Table 2:** Comparison of pain intensity (VAS, mm) and pressure pain thresholds (kg/cm^2^) values in the study groups.

	NIVR-G (*n* = 30)					CPT-G (*n* = 30)				
	Pre-Test	Post 10	Post 20	Post 30	Follow-Up	Pre-Test	Post 10	Post 20	Post 30	Follow-Up
Pain Intensity	46.67 ± 19.69	28.67 ± 16.39 *	30.93 ± 20.78	14.03 ± 8.24 *^,†^	32.03 ± 23.61	46.30 ± 24.11	33.67 ± 25.89	40.40 ± 327.43	39.47 ± 18.34	38.53 ± 26.57
PPT-T	3.72 ± 1.27	4.02 ± 1.21	3.90 ± 1.83	3.86 ± 1.09	3.57 ± 1.35	3.78 ± 1.14	3.16 ± 0.98	3.58 ± 1.32	3.26 ± 1.22	3.49 ± 1.12
PPT-K	3.90 ± 1.72	3.67 ± 1.11	3.96 ± 1.55	3.68 ± 1.21	4.03 ± 1.73	3.82 ± 0.87	3.32 ± 1.03	3.22 ± 1.28	3.33 ± 1.01	3.29 ± 1.22
PPT-H	4.14 ± 1.61	4.35 ± 1.46	4.46 ± 1.75	4.04 ± 1.58	4.06 ± 1.58	4.53 ± 1.67	3.87 ± 1.52	4.36 ± 1.56	4.21 ± 1.76	3.88 ± 1.53

Data expressed as means ± standard deviations; VAS Visual Analog Scale; PPT-T trapezius pressure pain threshold; PPT-K knee pressure pain threshold; PPT-H hip pressure pain threshold; * intragroup difference *p* < 0.05; ^†^ intergroup difference *p* < 0.05.

## Data Availability

All relevant data are presented in the manuscript. The datasets generated and/or analyzed during the current study are available from the corresponding author upon reasonable request.

## Supplementary Materials

The following supporting information can be downloaded at: https://www.mdpi.com/article/10.3390/medicina61071122/s1, Table S1: “Mean change in pain Visual Analog Scale scores according to patient-rated change”; Figure S1: “Minimum clinically important difference thresholds”.

## Abbreviations

The following abbreviations are used in this manuscript:

OA	Osteoarthritis
PPTs	Pressure pain thresholds
CS	Central Sensitization
VR	Virtual Reality
NIVR	Non-immersive virtual reality
CPT	Conventional physical therapy
VAS	Visual Analog Scale
MCID	Minimum clinically important difference
GRoC	Global Rating of Change
ACR	American College of Rheumatology
ACSM	American College of Sports Medicine
ANOVA	Analysis of variance
CPM	Conditioned pain modulation
