# Supplementary material for: Effects of Non-Immersive Virtual Reality Exercise on Self-Reported Pain and Mechanical Hyperalgesia in Older Adults with Knee and Hip Osteoarthritis: A Secondary Analysis of a Randomized Controlled Trial
Source: Medicina (Kaunas). 2025 Jun 21;61(7):1122. doi: 10.3390/medicina61071122 (PMC12298013; doi:10.3390/medicina61071122)
Supplement: Supplementary file 1 [file medicina-61-01122-s001.zip › Supplemental Online Material S1.pdf]

Supplemental Material I (Table S1)

Mean change in pain visual analog scale scores according to patient-rated change.

**Change categories**	**n (%)**	**Mean change (95% CI)**
**Extent of change**		
Baseline	60	46.48 (40.85 to 52.12)
No change	3 (5)	40.67 (-2.10 to 83.43)
A little better	7 (11.7)	34.71 (16.46 to 52.97)
Somewhat better	11 (18.3)	38.91 (26.84 to 50.98)
Moderately better	3 (5)	35.33 (-29.46 to 100.13)
A good deal better	32 (53.3)	20.81 (14.78 to 26.85)
A great deal better	3 (5)	8.33 (-6.01 to 22.68)
A very great deal better	1 (1.6)	NA
**Absolute change categories**		
No change, *n*	3 (5)	40.67 (-2.10 to 83.3)
Minimal change*, *n*	18 (30)	37.28 (28.22 to 43.34)
Moderate change, *n*	35 (58.3)	22.06 (15.98 to 28.13)
Large change, *n*	4 (6.7)	10.00 (0.81 to 19.19)

n number; CI confidence interval; NA Not Applicable for n=1. *Used to define the anchor-based estimate of the minimal clinically important change (see main text for details).
