# Supplementary material for: Effects of Non-Immersive Virtual Reality Exercise on Self-Reported Pain and Mechanical Hyperalgesia in Older Adults with Knee and Hip Osteoarthritis: A Secondary Analysis of a Randomized Controlled Trial
Source: Medicina (Kaunas). 2025 Jun 21;61(7):1122. doi: 10.3390/medicina61071122 (PMC12298013; doi:10.3390/medicina61071122)
Supplement: Supplementary file 1 [file medicina-61-01122-s001.zip › Supplemental Online Material S2.pdf]

Supplemental Material II (Figure S1)

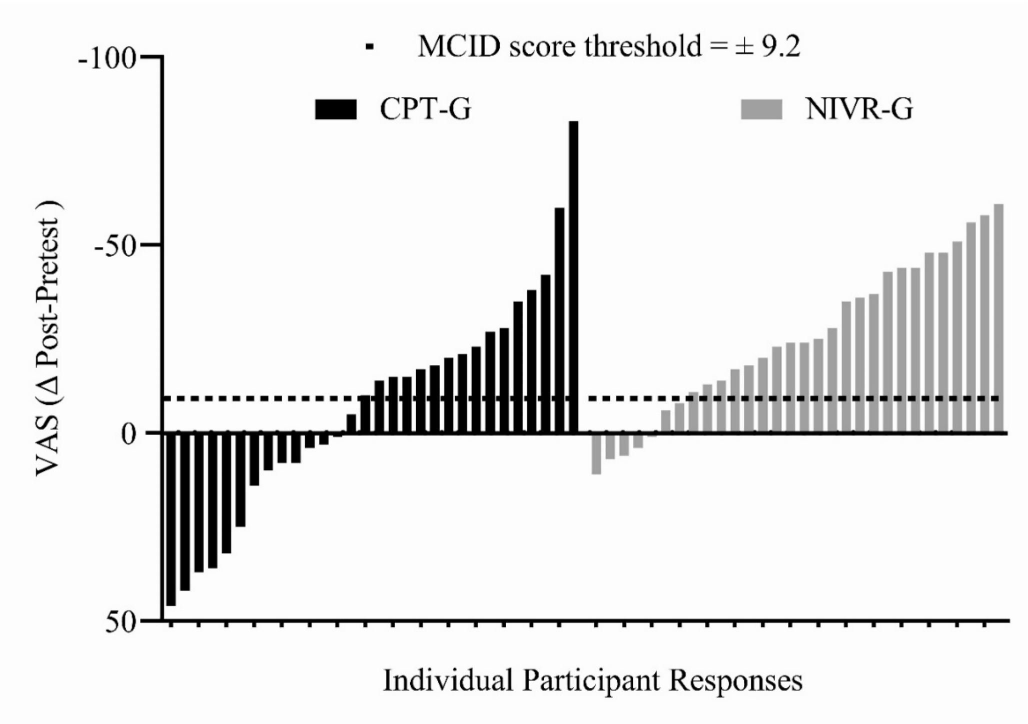

Minimum clinically important difference (MCID) thresholds for Visual Analog Scale (VAS) scores (horizontal line) and change (Δ) in each participant's scores (vertical bars) after 30 sessions of intervention, for classification as responders or non-responders. Conventional Physical Therapy group (CPT-G) and the Non-Immersive Virtual Reality group (NIVR-G).
